# Physical Activity as a Tool to Improve Sleep Quality for Secure Psychiatric Inpatients: A Feasibility Study

**DOI:** 10.1111/jsr.70095

**Published:** 2025-05-14

**Authors:** Poppy May Gardiner, Florence Emilie Kinnafick, Kieran C. Breen, Iuliana Hartescu

**Affiliations:** ^1^ School of Sport, Exercise, and Health Sciences Loughborough University Loughborough UK; ^2^ Research Centre St Andrew's Healthcare Northampton UK

**Keywords:** exercise, mental health, physical activity intervention, sleep quality

## Abstract

People with a severe mental illness (SMI) often experience insomnia and disrupted sleep–wake cycles. Daytime physical activity (PA) can retrain the sleep/wake cycle, but PA engagement is often markedly low in SMI. It is hypothesised that frequent, intermittent, short bouts of daytime PA can improve sleep outcomes in SMI. Twenty‐two inpatients from a secure psychiatric hospital (39.95 ± 16.87 years, 16 male) were recruited for a 10‐week PA intervention involving 3 × 10‐min bouts of self‐selected intensity PA/day, 5 days/week. Feasibility (primary outcome) was examined by assessing recruitment, outcome measure completion, trial adherence, and participant retention. At baseline, mid‐point, and post‐intervention, inpatients completed surveys, including the insomnia severity index (ISI) and wore the Motionwatch8 actigraph for 7 days to record sleep and PA data (including sleep efficiency (SE), total sleep time (TST), and moderate‐to‐vigorous PA (MVPA)). Outcome completion ranged from 67% to 91%. Mean trial adherence was 22% (range 2%–77%). Participant retention was 100%. Pre‐to‐post intervention, ISI scores decreased from 12.14 (SD = 4.98) to 11.38 (SD = 6.18) (*t* (20) = 0.65, *p* = 0.53). Time to bed and time of falling asleep both advanced (*p* > 0.05). Mid‐point of sleep (*M* = 38 min, SE = ≥ 15.93 min, *p* = 0.01) and wake time (*M* = 34 min, SE = 15.92 min, *p* = 0.02) significantly advanced. MVPA increased by 17 min/day (*T* = 1.17, *p* > 0.05). Other sleep indices (e.g., TST, SE) did not change significantly. Outcome completion and participant retention rates were high; trial adherence was low. Increasing intermittent PA can reduce psychiatric inpatients' insomnia symptoms and advance sleep phase. Sample variance and participant‐researcher rapport are discussed. Findings highlight the beneficial effect of short bouts of PA on sleep, providing scaffolding for future trials.

## 1 Introduction

Circadian rhythms, such as the sleep/wake cycle, are internally generated biological rhythms controlled by molecular mechanisms in the brain. Via exposure to external environmental light–dark cues, rhythms approximate to a 24‐h cycle of activity and rest, entrained to the day/night environment (Mistlberger and Skene 2005). These rhythms can also be entrained via timed behavioural cues such as mealtimes, social demands, and episodes of increased physical activity (PA). Indeed, human and animal models have consistently identified the chronobiotic properties of PA engagement (Mistlberger and Skene 2005). Further, short term daytime movement restriction—reflective of sedentary behaviour—can weaken circadian rhythms (Miyagi et al. 2019). Daytime sleep, or ‘napping’, can have a similar impact; altering the daytime‐accumulated pressure for nighttime sleep and delaying nighttime sleep and wake time the next morning (Monk et al. 2001).

Inactivity, sedentary behaviour, and napping are prevalent issues among inpatients in secure psychiatric care (Kinnafick et al. 2018; Rogers et al. 2021). It therefore follows that this population is at an increased risk of experiencing circadian misalignments such as a delayed sleep phase, in which bedtime and wake times are later than conventional sleep patterns (Evans and Hasler 2022). This misalignment is highly prevalent among people with severe mental illness (SMI) (Waite et al. 2018) and can often be comorbid with insomnia disorder (Evans and Hasler 2022), symptoms of which are also prevalent among secure psychiatric inpatients (Hartescu et al. 2022).

When targeting rhythm misalignment, PA can be implemented to delay or advance the onset of melatonin secretion; a hormone that precipitates the initiation of sleep (Buxton et al. 2003). PA can delay melatonin onset and sleep phase when taken during an individual's biological night‐time (Barger et al. 2004), or advance melatonin onset and sleep phase when taken during the morning, afternoon, and early evening (Buxton et al. 2003). Specifically, PA at 07:00 and between 13:00 and 16:00 advances melatonin onset, and PA taken between 19:00 and 22:00 delays melatonin onset (Youngstedt et al. 2019). In SMI, increased daytime PA can also serve to reduce insomnia symptoms and improve subjective sleep quality (Gardiner et al. 2024; Lederman et al. 2019). Breaking up sedentary behaviour with daytime PA can also improve daytime alertness (Rosenberger et al. 2019) which can reduce rhythm‐altering daytime sleep (Monk et al. 2001).

While it is evident that increased PA engagement can aid inpatient sleep quality, it is unclear how best to facilitate such behaviour change in this population, as institutional and individual barriers are prevalent and influential (Rogers et al. 2021). For example, ward restrictions due to risk presentation can prevent a patient from accessing off‐ward facilities such as the gym or swimming pool or outdoor areas like the hospital gardens and grounds for walking (Gardiner et al. 2024). Further, there is no optimal ‘dosage’ of PA engagement for this population (Deenik et al. 2017). While low intensity, short bouts of PA are recommended for tackling inactivity in habitually sedentary individuals with SMI (Deenik et al. 2017), it is not yet known whether this little and often ‘snacktivity’ model of PA engagement (Tyldesley‐Marshall et al. 2021) is feasible to implement and execute among psychiatric inpatients in secure care.

Moreover, randomised‐controlled trials (RCTs) in SMI often report low and highly varied rates of outcome measure completion, intervention adherence, and participant retention (MacInnes and Masino 2019; Tarrier et al. 2010). Marked variations in mental health and related cognitive capabilities within this population may influence these outcomes (Tarrier et al. 2010). As such, trials which promote patient‐led engagement and provide flexibility and tailoring to individual needs may help reduce study burden, improving outcome completion, adherence, and retention. Using a diverse set of research instruments (e.g., self‐report questionnaires and instrumental actigraphy) could also create more data collection opportunities for a wider range of inpatients, potentially achieving the same effect. However, these study tools have not yet been incorporated into ‘snacktivity’ based RCTs within this inpatient population.

Our overall aim was to better understand the feasibility of, and optimal methodological approach for, testing PA interventions to improve sleep and circadian outcomes in this under‐researched and vulnerable clinical population. Specifically, as markers of trial feasibility, we aimed to assess study recruitment, outcome measure completion, intervention adherence, and participant retention reporting for the 10‐week PA intervention. Further, it is not yet known whether successful execution of such a PA engagement model serves to improve sleep and circadian outcomes in this population. We therefore hypothesised that our 10‐week PA intervention would result in: (1) increased daytime PA; (2) decreased daytime sedentary time; (3) decreased daytime sleep; (4) a decrease in self‐reported insomnia symptom severity; and (5) an objective advance in sleep phase, as measured by actigraphy.

## 2 Methods

This feasibility trial was conducted at a secure psychiatric hospital in the UK. The University's Ethics Committee granted ethical approval (Reference: 2021‐5058‐4586). The Hospital's Research Peer Review Committee approved the research (Reference: 2021/130).

### 2.1 Study Design

The theoretical development and co‐design of this study are outlined in full detail in a previous publication (Gardiner 2023). The trial design was a repeated measures pre‐post trial, with seven‐day assessments at baseline, midpoint (week 5 of the 10‐week intervention), and post‐intervention. The 10‐week PA intervention commenced within 4 weeks of baseline assessment. Post‐intervention assessments were conducted within 6 weeks of intervention completion.

### 2.2 Recruitment and Participants

Hospital staff identified eligible inpatients based on the criteria in Table 1. Then, the first author contacted each patient to gain their informed consent. Earlier publications outline the recruitment procedures in full detail (Gardiner 2023; Gardiner et al. 2024).

**TABLE 1:** Participant eligibility criteria.

Inclusion criteria	Exclusion criteria
Adult inpatient (18 years or older).	Outpatients or individuals accessing the veteran's community care service.
Admitted to ward for 3 months or otherwise ‘stable’ on ward by WM or RC^a^ standard.	Inpatients in an acute state (i.e., acute depression, acute psychosis) at the time of data collection (confirmed by WM/RC).
Mental capacity to understand research information and informed consent.	Inpatients physically restrained, secluded, or given PRN^b^ (pro re nata medication) to de‐escalate hostile behaviour within the 48 h prior to data collection (confirmed by WM/RC).
Self‐reporting difficulty sleeping on the ReQOL‐20.^c^	Inpatients experiencing disordered eating or other conditions, for whom a physical activity intervention may be inappropriate.
Approved to partake by RC and WM.	
Forensic and non‐forensic care pathways.	

^a^ Ward Manager and Responsible Clinician.

^b^ PRN (as required medication).

^c^ Indicating sleep difficulty on the Likert Scale of the Recovering Quality of Life Scale (ReQOL‐20).

### 2.3 Procedure

At all three 7‐day assessments, participants were asked to complete self‐reported questionnaires (see secondary outcomes) and wear a waterproof wrist‐worn actigraph on their non‐dominant hand (Motionwatch 8 (MW8), CamNtech, Cambridgeshire, UK). Routinely collected hospital data (Table 2) were recorded at each period as described in full detail in earlier publications (Gardiner 2023; Gardiner et al. 2024).

**TABLE 2:** Participant demographic, medical, and incident information.

Outcome type	Specific outcome or measure
Demographic	Age Sex Gender Ethnicity Length of hospital admission Ward admission
Medical	Current primary ICD‐10 psychiatric diagnosis(es) Physical health disorder diagnosis(es) Body mass index (BMI) Mental Health Act Section for Admission Regularly Prescribed Psychiatric Medication PRN (pro re nata: administered as needed) psychiatric medication
Incident	Violence, self‐harm and suicide risk (HONOS A‐G and HONOS 1‐12)^a^ Recovering quality of life (ReQOL‐20) Incidence reporting (DATIX)^b^ Timing of routine patient observations^c^ Staffing requirements^d^ Leave availability (section 17)^e^

*Note*: Data were collected across all time points where possible. Due to significant missing or insufficient data in hospital records, it was not possible to present or analyse data for PRN medication, HONOS, ReQOL‐20, DATIX, or staffing requirement data. As such, these data are not referenced in the results.

^a^ Risk of engaging in verbal/physical aggressive/violent incidents, to others/self.

^b^ Actual verbal/physical aggressive/violent incidents, to others/self.

^c^ Mandatory nightly safety observations conducted by staff (constant or every 5, 15, 30, or 60 min).

^d^ No. of staff to be in a physical radius or line of sight of patient.

^e^ Patients' legal right to leave the ward, building, or hospital grounds.

### 2.4 Physical Activity (PA) Intervention

Participants engaged in a 10‐week PA intervention in which they were asked to complete 10‐min bouts of PA at three time periods daily, Monday to Friday. These three time periods (10:00–11:30, 13:30–15:00, 16:00–17:30) were identified following consideration of the existing circadian rhythm research, which recommends engaging in morning or afternoon PA to advance sleep phase and not facilitate possible phase delays that may result from evening exercise (Youngstedt et al. 2019). Practical consideration of hospital routine (meal and medication times, etc.) and ward staff availability was also considered. Participants were informed that PA of ≥ 2 min was encouraged where 10 min was not possible (Tyldesley‐Marshall et al. 2021).

Suggested activities, approved by the Hospital's Physiotherapy and Sport and Exercise teams, included (but were not limited to) walking, yoga, table tennis, and organised exercise group sessions. PA took place in ward spaces such as the outside courtyard area or hospital spaces such as the swimming pool. There was no PA coach or trainer; patients managed their own PA, self‐selecting their activity and intensity, under ward staff supervision. Participants were welcome to engage in PA alone, with staff, or with other patients. The first author regularly attended wards to monitor and encourage PA engagement and participated in activities when invited to by staff and patients but did not offer structured or regular one‐to‐one PA sessions. Participants and ward staff were given a brief PA adherence diary to complete during the intervention.

## 3 Primary Outcome: Feasibility

Study Recruitment: The number of available wards and potential, eligible, contacted, and recruited participants was recorded during both of the two recruitment waves. Reasons for eligible participants to not partake were also recorded.

Outcome Measure Completion: The number of participants completing each of the secondary outcome measures (listed below) was recorded at each time point (baseline, mid‐point, post‐intervention). Reasons for non‐completion were also recorded where known.

Intervention Adherence: Adherence to the 10‐week PA intervention was calculated (for *n* = 21) via a triangulation of inpatient completed adherence diaries, hospital records of patient PA (scheduled gym use, etc.), and raw actigraphy data. Adherence was calculated for each participant by considering how many sessions (of a maximum of 150) were engaged in across the 10‐week PA trial period. Using the median of adherence scores, patients were assigned to low‐adherer (*n* = 11) or high‐adherer (*n* = 10) groups in order to assess whether the secondary outcomes differed in the two groups at baseline assessment.

Participant Retention: Participant retention, presented as a percentage score, was calculated for the 10‐week PA intervention period. It is important to note that one male participant was discharged post‐baseline and did not begin the intervention. This participant's data is included in the description of the baseline sample (Tables 3 and 4); however, the data was *not* used in the subsequent pre‐post analyses.

**TABLE 3:** Participant demographics at baseline.

Sample characteristics	*n*	%	M	SD
Age	22		39.95	16.87
Sex (*n* = 22)				
Men	15	68.18		
Gender (*n* = 22)				
Male	16	72.72		
Ethnicity (*n* = 22)				
White (British, Irish, other)	20	90.90		
Mixed White and Black African	1	4.55		
Black or Black British Caribbean	1	4.55		
Physical health comorbidity (*n* = 15)				
Without comorbidities	1	6.66		
With comorbidities	14	93.33		
Body mass index (*n* = 14)				
Healthy weight	2	14.29		
Overweight to severely obese	12	85.71		
Primary psychiatric diagnosis^a^				
Schizophrenia/psychosis	10	38.46		
Mood disorder	2	7.69		
Personality disorder	13	50.00		
Developmental disorder	1	3.85		
Section 17 leave^b^ (*n* = 20)				
Medical appointments/building only	6	30.00		
Hospital grounds/local/home leave	14	70.00		
Nightly observations by staff^c^ (*n* = 22)				
Intermittent observations	19	86.36		
Constant observations	3	13.64		
Hospital admission duration (*n* = 22)				
0–12 months	9	40.91		
12–14 months	4	18.18		
24 months+	9	40.91		
Medication (*n* = 21)^d^				
Regularly prescribed antipsychotic	11	52.38		
Regularly prescribed antidepressant	5	23.81		
Regularly prescribed anxiolytic	3	14.29		
Regularly prescribed hypnotic	0	00.00		

^a^ Four patients reported multiple primary diagnoses.

^b^ Patients legal right to leave the ward, building, or hospital grounds.

^c^ Mandatory intermittent nightly safety observations conducted by hospital staff.

^d^ Two patients did not have a record of these medications being prescribed.

**TABLE 4:** Secondary outcomes at baseline.

Outcome	M	SD	Range^a^
Insomnia severity index (ISI)^b^ (*n* = 21)			
ISI total score	12.05	5.01	2–23
Night total	4.43	2.48	1–10
Day total	7.67	3.54	1–14
Actigraphy variables (sleep)^c^ (*n* = 19)			
Total sleep time (min)	431.42	106.47	
Sleep efficiency (%)	82.53	7.78	
Wake after sleep onset (min)	74.89	36.71	
Sleep onset latency (min)	12.84	19.83	
Daytime sleep/nap duration (min)	100.53	63.40	
Daytime sleep/nap frequency (nap/day)	3.68	2.52	
Actigraphy variables (activity)^b^ (*n* = 19)			
Daily light physical activity (min)	271.91	67.44	
Daily moderate‐vigorous physical activity (min)	190.98	89.56	
Daily non‐movement/sedentary time (min)	354.21	105.03	

^a^ At baseline.

^b^ Higher scores indicate more symptoms.

^c^ Data derived from a minimum of 3 continuous valid nights.

### 3.1 Secondary Outcomes

Participants were instructed to complete the insomnia severity index (ISI) at all three assessment periods (baseline, midpoint, post‐intervention). The ISI is a self‐reported questionnaire assessing the severity and impact of insomnia where higher scores are indicative of more severe symptoms (Morin 1993). The ISI creates a total score and can also be scored on two separate factors: night‐time and day‐time symptoms (Clemente et al. 2021). In a clinical sample, a change score pre‐to‐post treatment of −8.4 points (95% CI: −7.1, −9.4) has been associated with a moderate, clinically significant improvement to insomnia symptoms (Morin et al. 2011). The ISI has been identified as a viable sleep measure for psychiatric patients, as it discriminates between clinical and non‐clinical insomnia cases with good accuracy and high sensitivity in this population (Seow et al. 2018). The ISI has shown high internal consistency (α = 0.91) (Morin et al. 2011) and test–retest reliability (0.887, *p* < 0.01) (Clemente et al. 2021). In the current sample, at baseline, the Cronbach alpha for the total ISI was 0.60, indicating acceptable reliability of the instrument (Manzar et al. 2021).

Participants were also instructed to (1) wear the wrist‐worn MW8 continuously for an eight‐night/seven‐day period at all three assessment periods (baseline, midpoint, post‐intervention) and (2) identify all times they intended to fall asleep and all times of awakening by pressing the event marker button on the watch face. If not pressed, data were identified using the software's automatic identification algorithm and visually assessed. Actigraphy data were sampled in Mode 3, at 30 s epochs, analysed using MotionWare software (version 1.3.17, CamNtech Ltd.). Secondary outcomes derived from the MW8 included daytime sleep duration and frequency, night‐time sleep onset latency, wake after sleep onset, total sleep time (TST), and sleep efficiency. Five time points in the sleep/wake cycle phase were identified: time to go to bed, time of falling asleep, midpoint of sleep, time to wake up, and time to get out of bed (see LeMay‐Russell et al. 2021).

The MW8 recorded PA and non‐movement time (a marker of sedentary time) in counts per minute (CPM). Cut points were set as: moderate‐to‐vigorous PA (MVPA) ≥ 647 CPM, light activity = 107–646 CPM, and sedentary time < 107 CPM. Cut points were calculated by averaging the limited available literature on validated MW8 cut points from elderly (Landry et al. 2015) and young (Lin 2017) populations, using previously published methodology (Troiano et al. 2008). The valid files included for analyses comprised wear time of at least three nights over the measurement period (Troiano et al. 2008).

Demographic, medical, and incident data, including self‐harm and aggressive incidents, routinely collected from the hospital were securely retrieved for analysis (Table 2).

### 3.2 Data Analysis

Data were entered first into Microsoft Excel (Version 2207) then exported into SPSS (Version 27.0.1.0) for analysis. For efficacy testing, a predetermined analysis plan was implemented, dependent on the normality of data distribution; identified via histograms and statistical testing (Shapiro–Wilks). Two data points were identified via boxplots as statistically meaningful, influential outliers in moderate‐to‐vigorous PA and removed prior to analysis. Mauchly's test of sphericity indicated that the assumption of sphericity had not been violated. When data were normally distributed, repeated measures *t* tests, one‐way repeated measure ANOVAs, and chi squared tests were conducted. Wilcoxon signed rank test and Friedmans test were conducted when data were non‐normally distributed. Effect sizes (Cohen's *d*) are reported where available. For all analyses, statistical significance was defined at *p* < 0.05. Analysis was performed on the whole sample unless stated, with further completer analysis conducted where appropriate. Though a formal power calculation is typically not performed for feasibility testing, here, a power calculation was performed using G*Power based on the ISI (secondary outcome) to test the hypotheses outlined in the introduction regarding early efficacy testing. Using an expected effect size of *d* = 0.65, derived from a meta‐analysis of sleep interventions in secure psychiatric populations (Gardiner et al. 2022) and with *α* = 0.05 and *β* = 0.8, it was identified that a total of 21 participants were required for this trial.

### 3.3 Missing Data

For efficacy testing (secondary outcomes only), mean imputation, in which a group mean of the observed values for each variable is calculated, and the missing values for that variable are imputed by this mean, was conducted as necessary to handle missing data. This method for handling missing data has been seen in previous clinical trials with small sample sizes (see, Crooks et al. 2020). Mean imputation was conducted prior to pre–post analyses.

## 4 Results

### 4.1 Participants

A total of 22 inpatients (39.95 ± 16.87 year, 16 male) participated in baseline assessments (full description in Table 3). Participants were recruited from nine wards: two low secure, four medium secure, two medium to low transition wards, and one medium secure neurorehabilitation ward. Four participants recorded multiple primary diagnoses (e.g., comorbid schizophrenia and personality disorder). Sixteen participants were admitted from a forensic pathway. Hospital admission length ranged from five weeks to 10.5 years.

At baseline, the average ISI total score was 12.05 (SD = 5.01), indicative of subthreshold insomnia, and actigraphy data showed that the average TST was 431 min/night (SD = 106.47 min). Actigraphy data (from all times across the wake period, *not* restricted to allotted intervention session times) indicated that participants engaged in an average of 354 min/day (SD = 105 min) of non‐movement time (a marker of sedentary time), 272 min/day (SD = 67 min) of light PA, and 191 min/day (SD = 89 min) of moderate‐to‐vigorous PA (MVPA). Tables 3 and 4 illustrate all baseline secondary outcome data.

### 4.2 Adverse Incident Reporting

One participant experienced an ankle sprain during a routine PA session with the Hospitals' Occupational Therapy team. They received first aid and rested for 2 weeks. They remained in the study during that time and re‐engaged with PA when recovered.

### 4.3 Primary Outcome: Feasibility

#### 4.3.1 Study Recruitment

Recruitment occurred in two waves (full description in Table 5). In Wave One, from 86 potential participants, 28 (33%) fit the eligibility criteria, 20 (23%) were contacted, and finally 17 (20%) were recruited. In Wave Two, from 30 potential participants, six (20%) were eligible and contacted, and three (10%) were recruited. Though not formally recorded, most potential participants were deemed ineligible by their supervising clinical team due to either heightened risk presentations or being unable to give informed consent due to being in an acute mental state. Four individuals were deemed ineligible for reasons outside the established exclusion criteria: two people were deaf and not accompanied by a full‐time interpreter, and two were wheelchair users and deemed by hospital clinicians to be unable to partake in the PA regularly as required by the trial. Additionally, four eligible people were not present or available during a number of concurrent study visits. Across both recruitment waves, six eligible contacted participants were not interested in partaking and were not recruited. After Wave Two, hospital clinicians identified two additional eligible participants from a Wave One ward. These individuals were contacted and later recruited by the first author.

**TABLE 5:** Study recruitment progression.

Wave	Wards	Potential participants	Eligible participants (*n*) (%)	Contacted (*n*) (%)	Recruited (*n*) (%)
1	6	86	28 (33%)	20 (23%)	17 (20%)
2	2	30	6 (20%)	6 (20%)	3 (10%)

*Note*: (%) is of the ‘Potential participants’ at all time‐points.

#### 4.3.2 Outcome Measure Completion

Table 6 shows the completion rate of secondary outcome measures. Though not formally recorded, prominent reasons for non‐completion were individual or ward isolation for COVID‐19, or clinicians deciding the wrist‐worn actigraphy was not appropriate for that participant.

**TABLE 6:** Secondary outcome measures completed.

Time	Insomnia severity index (Morin 1993) (*n*) (%)	Wrist‐worn actigraphy (*n*) (%)
Baseline (max. *n* = 22)	21 (96%)	19 (86%)
Mid‐point (max. *n* = 21)	17 (81%)	13 (62%)
Post‐intervention (max. *n* = 21)	20 (95%)	14 (67%)
All time points (max *n* = 64)	58 (91%)	43 (67%)

*Note*: One participant was discharged from the hospital prior to the intervention beginning.

#### 4.3.3 Intervention Adherence

Intervention adherence, measured via adherence diaries, hospital records, and actigraphy data (outlined in Section 2) ranged from 2% to 77%, with a mean of 22% of PA sessions engaged in, from a maximum of 150 potential PA sessions across the 10‐week intervention period (3 sessions daily for 5 days a week). There were no significant differences between low and high adherer groups at baseline (Table 7).

**TABLE 7:** Baseline statistical differences between low‐adherers and high‐adherers.

Outcome	*p*	Test statistic	df
Insomnia severity index (ISI) total	0.07	−0.57^a^	19
ISI day	0.18	−1.58^a^	19
ISI night	0.08	1.04^a^	19
Daytime sleep/nap duration^c^ (min)	0.81	0.79^a^	17
Daytime sleep/nap frequency^c^ (naps/day)	0.44	0.94^b^	17
Sleep onset latency^c^ (min)	0.10	−0.72^b^	17
Wake after sleep onset^c^ (min)	0.85	0.24^b^	17
Total sleep time (night)^c^ (min)	0.81	1.01^b^	17
Sleep efficiency^c^ (%)	0.55	0.62^a^	17
Non‐movement/sedentary time^c^ (min)	0.79	0.37^a^	17
Light physical activity^c^ (min)	0.43	−0.26^b^	17
Moderate‐vigorous physical activity^c^ (min)	0.93	−1.83^b^	17

^a^ Significance identified via parametric testing.

^b^ Significance identified via non‐parametric testing.

^c^ Actigraphy data derived from a minimum of an average of three continuous valid nights of monitoring.

#### 4.3.4 Participant Retention

Across the 10‐week PA intervention period, participant retention was 100%. Moreover, no participant withdrew from the study at any time, nor were any asked to leave the study at any time by either hospital staff or the research team.

### 4.4 Secondary Outcomes

#### 4.4.1 Insomnia Severity Index

There was a non‐significant decrease in total ISI scores from 12.14 (SD = 4.98) at baseline to 11.38 (SD = 6.18) post‐intervention (*t* (20) = 0.65, *p* = 0.53, *d* = 0.14), with a mean difference of 0.76. There were no significant changes in total ISI scores between any of the three assessment points (*F* (2,40) = 1.37, *p =* 0.27). There were no significant changes in daytime ISI symptoms (*F* (2,40) = 2.68, *p =* 0.08) nor nighttime ISI symptoms (*F* (2,40) = 0.74, *p =* 0.48) between any of the three assessment points. There was no significant effect of intervention adherence on total ISI change scores from baseline to post‐intervention (*t* (19) = −0.13, *p* = 0.90).

To determine the clinical significance of the change in total ISI score, a Chi Squared analysis was completed, where the total ISI result was categorised as either sub‐threshold insomnia (< 14) or moderate to severe insomnia (≥ 15) (Morin et al. 2011). Analysis indicated no significant change in rates of remission from baseline to post‐intervention (*χ* ^*2*^ (1,21) = 0.47, *p =* 0.08).

Females (*n* = 5) reported a higher mean ISI score than males (*n* = 16) throughout. At baseline, females scored 14.40 (SD = 5.98) and males scored 11.44 (SD = 4.62). At midpoint, females scored 17.46 (SD = 6.61) and males scored 11.99 (SD = 5.65). At post‐intervention, females scored 11.60 (SD = 5.55) and males scored 11.31 (SD = 6.53). Robust statistical testing on these differences was not possible due to sample heterogeneity. The trend is explored in the Section 5.

### 4.5 Actigraphy Derived Sleep/Wake Cycle Outcomes

Non‐parametric testing revealed that from baseline to post‐intervention, there was a non‐significant mean (average) advance in time to go to bed of 37 min (*T* = −1.29, *p* = 0.20, SE = 15.93 min), a non‐significant mean advance in time of falling asleep of 40 min (*T* = −1.54, *p* = 0.12, SE = 15.93 min), a significant mean advance in the midpoint of sleep of 38 min (*T* = −2.76, *p* = 0.01, SE = 15.93 min), and a significant mean advance of 34 min in both time to wake up (*T* = −2.39, *p* = 0.02, SE = 15.92 min) and time to get out of bed (*T* = −2.27, *p* = 0.02, SE = 14.31 min). Table 8 illustrates this data.

**TABLE 8:** Actigraphy derived sleep/wake cycle outcomes.

Activity	Baseline (*n* = 19)		Mid‐point (week 5) (*n* = 13)		Post‐intervention (*n* = 14)	
	Mean time	SD (min)	Mean time	SD (min)	Mean time	SD (min)
Time to get into bed	23:20	122.86	23:01	133.81	22:43	94.28
Time of falling sleep	23:33	113.44	23:10	129.95	22:53	97.68
Midpoint of sleep^a^	03:47*	90.55	03:20	119.16	03:09*	87.30
Time to wake up	08:01**	101.22	07:29	145.14	07:27**	113.18
Time to get out of bed	08:04***	101.37	07:32	145.32	07:30***	113.03

*Note*: *, **, ***Significant difference from baseline to post‐intervention.

^a^ Representative of clock time between sleep onset and waking up.

### 4.6 Actigraphy Derived PA

Moderate‐to‐vigorous PA (MVPA) across the wakeful period, not restricted to allotted adherence times, increased by 17 min/day, from 191 min/day (SD = 89 min) at baseline, to 208 min/day (SD = 101 min) post‐intervention, though this was non‐significant (*T* = 1.17, *p* = 0.24). MVPA significantly increased by 51 min/day, from 157 min/day (SD = 63.51 min) at midpoint, to 208 min/day (SD = 101.14 min) post‐intervention (*T* = −2.35, *p* = 0.02). In considering the impact of adherence, from baseline to post‐intervention, the low‐adherer group MVPA increased by an average of 13 min/day, whereas the high‐adherer group MVPA increased by an average of 20 min/day. This difference was non‐significant (*T* = 1.63, *p* = 1.02).

Light PA across the wakeful period, not restricted to allotted adherence times, significantly decreased by 40 min/day, from 272 min/day (SD = 67.44 min) at baseline to 232 min/day (SD = 35.78 min) post‐intervention (*T* = −2.63, *p* = 0.01). However, there was a significant difference (*T* = 2.47, *p* = 0.01) in light PA at post‐intervention between the high‐adherer group (M = 253 min/day, SD = 25.45 min) and the low‐adherer group (M = 213 min/day, SD = 33.36 min). In the high‐adherer group, the decrease in light PA from baseline to post‐intervention was not significant (*T* = −0.77, *p* = 0.44), with average light PA decreasing by 21 min/day, from 149 min/day (SD = 409 min) at baseline to 128 min/day (SD = 396 min) post intervention. In contrast, in the low‐adherer group, the average light PA decreased by 50 min/day, from 153 min/day (SD = 386 min) at baseline to 103 min/day (SD = 366 min) at post‐intervention, which was significant (*T* = −2.60, *p* = 0.01).

### 4.7 Other Actigraphy Derived Sleep and Movement Variables

There were no significant changes in daytime sleep duration and frequency, sleep onset latency, wake after sleep onset, total sleep time, or sleep efficiency across the three assessment periods. There was a non‐significant increase in non‐movement time, as a marker of sedentary behaviour, from 354 min/day (SD = 105.03 min) at baseline to 365 min/day (SD = 99.77 min) at post‐intervention (*t*(18) = 0.58, *p* = 0.57, *d* = 0.13). There were no significant changes in non‐movement time between any of the three assessment points (*F* (2,36) = 0.34, *p* = 0.57).

## 5 Discussion

From the admitted inpatients, across two recruitment waves, only a small percentage were eligible (20%–33%), contacted (20%–23%), and recruited (10%–20%) for this study. Most outcome measures were completed by most participants. Intervention adherence was low; however, the participant retention rate was 100%. From baseline to post‐intervention, on average, there was a reduction in self‐reported insomnia symptoms, a significant advance in actigraphy‐derived sleep phase, and an increase in moderate‐to‐vigorous PA. There were no statistically significant differences in total sleep time, sleep efficiency, sedentary time, or daytime sleep.

### 5.1 Feasibility: Study Recruitment

Eligibility ranged from 20% to 33% across the two recruitment phases. This is within an expected range, given other recent feasibility trials with psychiatric inpatients have seen rates closer to ~15% (Kleiman et al. 2024). Although not specified as exclusion criteria pre‐commencement of study procedures, people who are deaf and people who are wheelchair‐bound did not participate in this research. As such, this study cannot conclude that the trial protocol is feasible for these subgroups, and future research would be needed to develop feasible protocols for these individuals.

Following eligibility screening, recruitment ranged from 50% to 61% across the two recruitment phases; again, comparable to similar trials in the field that reported ~61% recruitment success (Kleiman et al. 2024). Many eligible participants in the current trial were simply not interested in partaking (*n* = 4) or were not available during study visits (*n* = 6), which is common when working with this clinical population (Kleiman et al. 2024). Due to the significant fluctuations in mental health status and mood seen in this population, the levels of reported disinterest in this study are expected. Similarly, with many patients taken off‐ward to attend routine medical appointments and therapy sessions, the number of patients unavailable during study visits is also understandable. Future trials may explore the benefit of utilising a larger recruitment team to increase researcher availability and presence on the wards to facilitate more patient contact and recruitment options.

### 5.2 Feasibility: Outcome Measure Completion

Although missing data typically poses a challenge in inpatient mental health research (Radtke et al. 2024) self‐report surveys were completed in 91% of cases in the current work. Compared to completion rates of just 40%–67% as seen in other similar trials (Martland et al. 2024), this is substantial. It is well established that participant engagement is known to support data collection (Radtke et al. 2024). As the participants in the current work were highly engaged with this project—primarily as they had co‐designed it (Gardiner 2023; Gardiner et al. 2024)—this high completion rate is fitting. The importance of engagement and participant‐researcher rapport is explored further in an upcoming section of this discussion.

The wrist‐worn actigraphy device was worn in just 67% of cases; a rate more aligned to that of previous trials (Martland et al. 2024). Often, the device was not worn as hospital clinicians had deemed the participant to be presenting with heightened risk behaviours. Therefore, it was not suitable to provide them with an instrument that contained metal components. With so many adverse incidents seen in this population, especially among those with disturbed sleep (Hartescu et al. 2022), such risk management in a sleep quality intervention was absolutely necessary, despite the impact on outcome measure completion.

Considered together, these completion rates highlight the value of utilising both instrumental measures and self‐report measures for data collection in this clinical population; something which should be taken into consideration when designing future RCTs in this field.

### 5.3 Feasibility: Intervention Adherence

Intervention adherence is a vital aspect of PA research in SMI populations (Rosenbaum et al. 2014). In the current study, individual adherence to the PA sessions ranged from 2% to 77%, with a mean adherence score of 22% from a maximum of 150 sessions; similar to rates identified in previous PA trials in SMI populations (Bonsaksen 2011; Martland et al. 2024). COVID‐19 protocols were impactful to adherence rates, and this is discussed in greater detail in the Limitations section. Future trials could explore the possibility of reducing the 10‐min bout to 2 min, as this has been identified as acceptable and achievable in other highly sedentary populations (Tyldesley‐Marshall et al. 2021). Further, specifying an intensity of activity may be complementary to reducing the required time of engagement (Islam et al. 2022). Exploring the impact of such design changes on intervention adherence would be a useful extension to this field of research.

As illustrated in Tables 3 and 4, and indeed noted in previous work with this patient group (Gardiner et al. 2024), there was marked variance across participant data. This can be seen in the varying diagnoses, lengths of stay, freedom of movement within and outside of the hospital grounds, medication use, and baseline sleep, PA, and sedentary time outcomes. With such considerable heterogeneity among the participant group, the variance seen across intervention adherence scores (2%–77%) is not surprising. To consider just one source of variability—freedom of movement—it is possible that those with less freedom had fewer opportunities to engage in PA and so lower adherence scores. Comparatively, those with more freedom may have had more PA opportunities and, as a result, higher adherence scores. However, due to the small sample size in the current trial, it was not possible to conduct robust testing to examine the impact of this baseline variance statistically, as is discussed further in the Limitations section. While this study did follow the gold‐standard procedure of co‐designing the project with key stakeholders (Gardiner 2023), it is possible that more work needs to be done during the study design process to maximise the potential for tailoring the PA intervention to each individual; accounting for their baseline diagnosis, length of stay, freedom of movement, medication use, sleep health, PA engagement, and sedentary time.

### 5.4 Feasibility: Participant Retention

The current trial maintained a 100% retention rate across the 10‐week PA intervention. This is markedly high compared to both inpatient trials reporting 62% retention (Martland et al. 2024) and even *outpatient* trials reporting ~84% retention, on average (Stubbs et al. 2016). As strong therapeutic relationships can influence treatment adherence among inpatients (Fagan et al. 2021), the 100% retention rate may be due to the strong working relationship between the first author (lead‐researcher) and the participants. In total, 62% of the participants had previously partaken in interviews with the first author as a part of the project's co‐development (Gardiner et al. 2024). As such, the majority of the current sample were already familiar with the project and the research team before enrolling in the intervention. While this supports the notion that researcher‐author rapport was positively influential, this is just speculative.

Retention may also be due to the intermittent short bouts of PA being accessible, as has been identified in other sedentary groups (Tyldesley‐Marshall et al. 2021). Additionally, no significant adverse incidents arose from engaging in PA this way. This may also have served to promote and maintain intervention adherence.

### 5.5 Insomnia Symptoms

A non‐significant decrease in self‐reported insomnia symptom severity was seen from baseline to post‐intervention. Previous interventions with psychiatric inpatients have identified higher baseline ISI scores than the current sample, perhaps providing an increased opportunity to see a statistically significant decrease in score post‐intervention (e.g., Sheaves et al. (2018)).

Demographic differences may also have been influential to the ISI scores, as the majority of the study sample identified as male (Table 3). Males with SMI are significantly less likely to meet diagnostic criteria for insomnia than females (Zhu et al. 2022) and male inpatients are also more likely to benefit from sleep quality interventions than females, as are older inpatients compared to younger inpatients (Gardiner et al. 2022). As such, the relatively low ISI baseline and absence of significant change in insomnia symptoms pre‐to‐post may be due to the predominantly male cohort. However, due to this homogeneity, it was not feasible to conduct robust statistical tests to analyse the impact of gender or age in the current work. Future trials with larger and more diverse samples would be well placed to do so. This would be especially useful as the prior research from Sheaves et al. (2018) included only or predominantly males, leaving females largely understudied.

### 5.6 Sleep/Wake Cycles

There was a significant advance towards earlier awakening and time to get out of bed, as well as a significantly earlier mid‐point of sleep. Similarly, there was a non‐significant trend towards an earlier time to go to bed and time of falling asleep. With circadian misalignment associated with negative mental health consequences (Murray et al. 2017), shifts towards an advancement in this phase could be of considerable clinical benefit to the inpatient population. To the authors' knowledge, the current work is the first to demonstrate a gradually shifting phase advance following a 10‐week PA intervention in secure psychiatric inpatients. As circadian entrainment properties of PA depend on regular engagement (Van Someren and Der Riemersma‐Lek 2007) future studies may wish to extend the post‐intervention follow‐up measures to investigate the effect longevity of the 10‐week intervention, or extend the intervention period itself.

### 5.7 PA and Sedentary Time

The average volume of wrist‐worn actigraphy‐derived moderate‐to‐vigorous PA (MVPA) was 185 min/day; substantially higher than hip‐worn accelerometer‐derived data from outpatient (Chapman et al. 2016) and inpatient (Fraser et al. 2016) SMI populations, who reported 26 and 37 min/day, respectively. This high volume may be an indirect result of reduced night‐time sleep periods which resulted in participants having markedly long wake periods during the day; increasing the opportunities to engage in PA.

The wear location of the device may have been influential as wrist‐worn monitors (used here) can overestimate MVPA, compared to hip‐worn monitors (Gall et al. 2022). However, the consistent application of the same methods at all measuring points did ensure consistency of error, if any present, in the tools used. The wear location may also have been influential to the sedentary time recorded of 6 h 9 min/day; markedly low compared to hip‐worn data from inpatient SMI populations who reported 11.1 h/day (Fraser et al. 2016). As wrist‐worn devices are susceptible to over‐estimating PA (Gall et al. 2022), it is possible that this can result in a decrease in identified sedentary time. To be assessed more accurately in future research, postural components of sedentary behaviour would need to be measured, ideally by gold standard inclinometry (Chauntry et al. 2022).

The cut‐points used to derive actigraphy‐derived PA intensity and sedentary time were modelled from established published methodology (Troiano et al. 2008), based on averaging older and younger populations cut‐points (Landry et al. 2015; Lin 2017). There were no established cut points appropriate for the current SMI population, highlighting the need for the development of research in this area.

### 5.8 Daytime Sleep

Patients were seen to spend an average of 106 min/day engaging in daytime sleep; higher than adults in the community who self‐report between 41 and 50 min/day (Léger et al. 2019). Daytime fatigue, drowsiness, and antipsychotic and antidepressant medications—both commonly prescribed in this population (Hartescu et al. 2022)—can impact the likelihood of an inpatient with SMI engaging in daytime sleep (Rogers et al. 2021); a behaviour which can contribute to the weakening of the day/night rhythm by decreasing nighttime sleep pressure (Monk et al. 2001). More research is needed to test this in SMI, as only one other inpatient study has reported daytime sleep, though the methodology was unclear (Talih et al. 2018).

### 5.9 Other Actigraphy‐Derived Sleep/Wake Variables

There were no significant changes seen in total sleep time, sleep efficiency, sleep onset latency, and wake after sleep onset. When behaviour change interventions are multifactorial and underpinned by multiple theoretical techniques, as the current study is (Gardiner 2023), it is not uncommon for some elements of the trial to not be received as intended, which can decrease effectiveness (Brierley et al. 2022). As discussed earlier in this section, significant variation between participants (highlighted in Tables 3 and 4) may also have been influential here.

### 5.10 Strengths

The current sample is typical of those residing in inpatient psychiatric settings; therefore, the recruitment rates, outcome completion, intervention adherence, and participant retention figures could be used as guidance for future work in this area. Although behavioural interventions, including those which utilise PA, have been identified as the most effective for improving sleep quality in this population (Gardiner et al. 2022), the interactions between PA and sleep in this population had not yet been well explored (Rogers et al. 2021). Thus, the novel nature of the intervention itself is advantageous, as to the authors' knowledge, this is the first intermittent PA intervention to improve sleep quality in secure psychiatric care. Further, the intervention was co‐designed with hospital staff and inpatients and developed within a theoretical framework (Gardiner 2023), aligning to best practice guidelines from the UK Medical Research Council and the National Institute for Health and Care Research (Skivington et al. 2021). This design allowed the first author to visit the hospital regularly and spend time building rapport with inpatients and staff; a crucial aspect of working in inpatient care (Fagan et al. 2021). Further, as cognitive difficulties may make self‐report measures challenging for this population, adding the wrist‐worn monitor, which did not rely on cognitive engagement, was imperative (Fraser et al. 2016). The dual approach of implementing self‐reported and device‐based measures was a key strength of the work.

### 5.11 Limitations

Participants completed the intervention at different times in the year from September 2021 to May 2022. As such, seasonal changes relating to light exposure may have affected sleep schedules (Shochat et al. 2019); however, light exposure was not a measured outcome. Similarly, PA taken outdoors can provide greater benefits to mental health and wellbeing compared to PA taken indoors (Kajosaari and Pasanen 2021), but the location of PA was not recorded. The cut‐points were derived from general population studies as described. As secure psychiatric inpatients are known to be habitually sedentary (Rogers et al. 2021), have high rates of obesity (Huthwaite et al. 2017), and be heavily medicated (Hartescu et al. 2022), it stands that this sample would significantly differ from the community‐dwelling general population. Though adequately powered to conduct hypothesis testing, the sample size lacked power to conduct exploratory analyses to investigate the effect of any confounding variables such as gender, age, freedom of movement, medication, daytime sleep, PA engagement, or sedentary time. Larger sample sizes in future studies are recommended. National and hospital‐specific COVID‐19 protocols intermittently resulted in patient and ward isolations, low staffing numbers, and restricted ward access to the authors. As such, not all activities (e.g., leaving the ward to use the swimming pool) were accessible, staffing was too low to facilitate additional activities, and not all research procedures and visits could be conducted as scheduled.

## 6 Conclusion

It is feasible to increase overall PA among psychiatric inpatients using a little and often ‘snacktivity’ model of engagement. Eligibility criteria for such a programme of work need further consideration to increase accessibility, and the intricacies of the study design should be studied to support overall adherence. Future research may wish to consider using a varied set of outcome measures to support fruitful data collection. The value of participant‐researcher rapport should be prioritised when considering ways to support participant retention. Increasing intermittent PA engagement can significantly advance sleep phase and reduce insomnia symptoms. This trial provides the scaffolding for future research with larger samples to further explore the effects of PA on insomnia and explore the impact of trial duration on the longevity of the identified phase advance. This work extends what is currently a limited field of research; behavioural interventions to improve sleep quality, and offers, to the authors' knowledge, the first account of testing an intermittent PA intervention to improve sleep quality in secure psychiatric inpatients.

## Author Contributions

**Poppy May Gardiner:** conceptualization, data curation, formal analysis, methodology, project administration, validation, visualization, writing – original draft, writing – review and editing, investigation. **Florence Emilie Kinnafick:** funding acquisition, conceptualization, methodology, project administration, writing – review and editing, supervision. **Kieran C. Breen:** conceptualization, funding acquisition, methodology, writing – review and editing, project administration, supervision. **Iuliana Hartescu:** funding acquisition, conceptualization, data curation, formal analysis, methodology, project administration, validation, visualization, writing – review and editing, supervision.

## Ethics Statement

St. Andrew's Healthcare Research Centre Peer Review Committee approved the conduct of research in June 2021 (Reference: 2021/130). Loughborough University's Ethics Committee granted ethical approval for this research in July 2021 (Reference: 2021‐5058‐4586). All participants provided written informed consent prior to enrolment in the study.

## Conflicts of Interest

The authors declare no conflicts of interest.

## Data Availability

The data that support the findings of this study are available on request from the corresponding author. The data are not publicly available due to privacy or ethical restrictions.
